# Surgical treatment of papillary fibroelastoma of the pulmonary valve: a case report

**DOI:** 10.1186/s13019-022-01909-4

**Published:** 2022-06-09

**Authors:** Hiroo Uehara, Masateru Uchiyama, Takayuki Hori, Mitsuru Iida, Tomohiro Imazuru, Tomoki Shimokawa

**Affiliations:** 1 Department of Cardiovascular Surgery, Teikyo University, 2-11-1 Kaga, Itabashi-ku, Tokyo, 173-8605 Japan; 2 Department of Cardiovascular Surgery, The Cardiovascular Institute, Tokyo, Japan

**Keywords:** Papillary fibroelastoma, Pulmonary valve

## Abstract

**Background:**

Cardiac papillary fibroelastoma (PFE) is a rare tumor, and especially rare when found on the pulmonary valve.

**Case presentation:**

We report the case of a 70-year-old woman patient with a pulmonary valve PFE diagnosed incidentally during a follow-up of aortic regurgitation. Computed tomography and magnetic resonance imaging showed no suggestive signs of malignant tumors, and thrombus or myxoma was initially suspected. However, an initial transthoracic and transesophageal echocardiogram did not exclude the possibility of a malignant tumor attached to the wall of the pulmonary artery. Considering the embolization risk, we opted to perform tumorectomy, in which additional surgical procedures could then be conducted if intraoperative diagnosis showed a malignant tumor. Indeed, intraoperative findings showed the tumoral mass attached on the left semilunar cusp of the pulmonary valve, and intraoperative diagnosis of the tumor showed no malignancy. Planned tumorectomy was performed concomitantly with AVR. The pathologic examination of the removed tumor confirmed the diagnosis of PFE. Her postoperative course was uneventful without any sign of recurrence.

**Conclusion:**

This case highlights the difficulty of accurate diagnostic imaging and provides valuable insight into a successful surgical treatment of pulmonary valve PFE without any complications.

## Background

Cardiac papillary fibroelastomas (PFEs) are benign tumors of the endocardium, which account for 7–8% of all cardiac tumors [1]. Cardiac PFEs mainly originate in the left heart valve, but in very rare cases they have been found on the pulmonary valve [2]. However, as literature regarding the management of PFE is currently limited in itself, there is even less consensus on that of right-sided PFE. Here, we report a case of undiagnosed PFE on the pulmonary valve without any neurological and cardiovascular complications.

## Case presentation

**Fig. 1:**
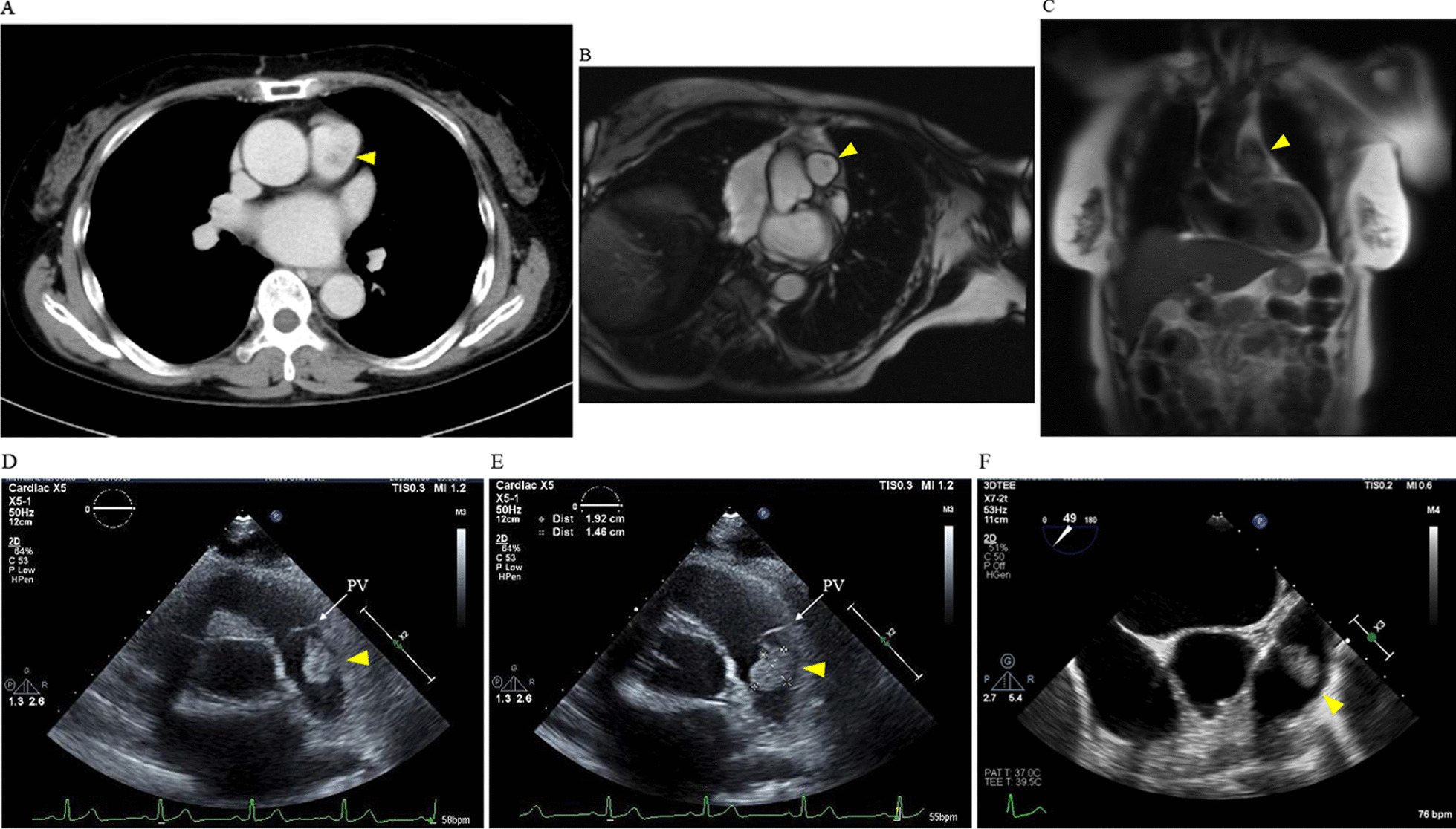
Preoperative findings. **A** Computed tomography. **B**, **C** Magnetic resonance imaging. **D**, **E** Transthoracic echocardiography. **F** Transesophageal echocardiography. (Each examination revealed a tumor shown as a yellow triangle)

A 70-year-old female during a follow-up of moderate aortic regurgitation (AR) was transferred to our institution for further investigation of an undiagnosed cardiac tumor around the pulmonary artery. She had a past medical history of hypertension and hyperlipidemia and was a former smoker. Initial physical examination findings showed stable hemodynamic values and a diastolic murmur at the left sternal border. Laboratory values on admission showed all chemical parameters including D-dimer, soluble fibrin monomer complex, interleukin-2, and tumor markers were within the normal range. Electrocardiogram was unremarkable. An initial computed tomographic (CT) scan revealed an irregular tumor (17 × 14 × 8 mm) located on the wall of the pulmonary artery (Fig. 1A). In addition, although the CT scan showed the approximate location of the tumor, it was limited with regards to differential diagnosis and identification of the stalk of the tumor. Gallium-67 scintigraphy imaging showed no suggestive signs of malignant tumors. Cardiac magnetic resonance imaging (MRI) showed that the tumor was located immediately above the pulmonary valve (Fig. 1B and C). Transthoracic echocardiography (TTE) showed a pedunculated mobile tumor located on the wall of the pulmonary artery (19 × 15 mm) and normal right ventricular systolic function with no pulmonary hypertension (Fig. 1D and E). TTE also showed normal left ventricular systolic function with an ejection fraction of 69% and moderate AR with vena contracta of 4 mm and pressure half time of 495 ms. Transesophageal echocardiography (TEE) showed a highly-mobile, pedunculated, and inhomogeneous 16 mm round tumor on the pulmonary artery, which was located away from the pulmonary valve (Fig. 1F). Because of no malignant findings shown in the blood test, TTE, TEE, and Gallium-67 scintigraphy imaging, thrombus or myxoma was initially suspected. In addition, the above preoperative examinations could not delineate clearly the relationship of the tumor with the pulmonary valve or the wall of the pulmonary artery. Considering the embolization risk, we reasoned that aortic valve replacement (AVR) and tumorectomy should be performed, and additional surgical procedures such as pulmonary valve replacement and graft replacement of pulmonary artery could then be conducted if intraoperative diagnosis showed a malignant tumor.

**Fig. 2:**
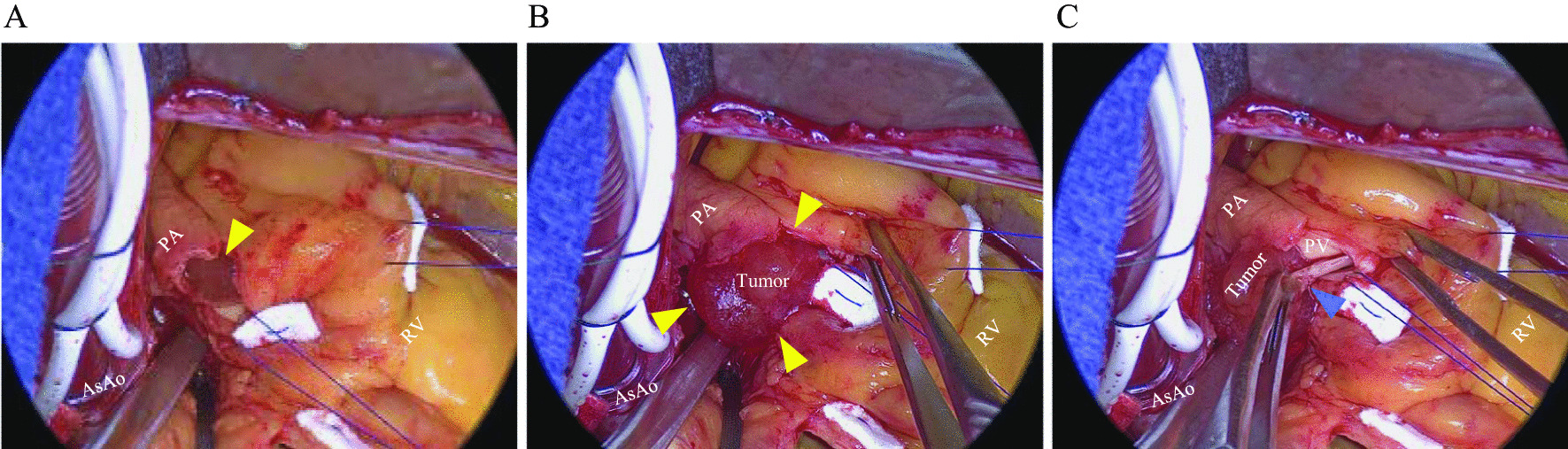
Intraoperative findings. **A** After incision of the pulmonary artery. **B**, **C** A 20 mm round tumor was confirmed (**B**) (shown as yellow triangles) and was attached on the left semilunar cusp of the pulmonary valve (**C**) (shown as a blue triangle). AsAo, ascending aorta; PA, pulmonary artery; RV, right ventricle; PV, pulmonary valve

**Fig. 3:**
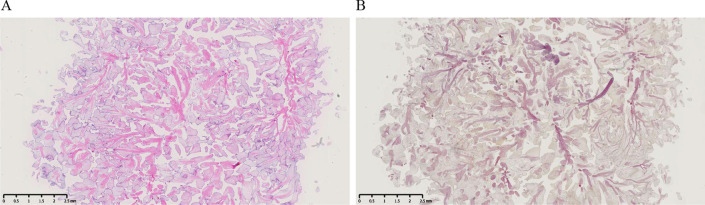
Pathological assessment. **A**, **B** A representative histological section of the tumor showed a benign papillary lesion comprised of a single layer of endocardial cells (**A**) and papillary fronds consisting of collagen and elastin fibers (**B**)

The procedure was performed under general anesthesia. Cardiopulmonary bypass was performed through cannulations of the ascending aorta and right atrium. After incision of the pulmonary artery, a 20 mm round tumor was discovered (Fig. 2A and B), which was attached on the left semilunar cusp of the pulmonary valve (Fig. 2C). Intraoperative diagnosis of the tumor showed no malignancy. Planned tumorectomy was performed concomitantly with AVR. Operation time, cardiopulmonary bypass time, and aortic cross-clamp time was 188, 120, and 91 min, respectively. The pathologic examination of the removal tumor confirmed the diagnosis of PFE that originated from the pulmonary valve. Hematoxylin–eosin staining of the tumor showed a benign papillary lesion comprised of a single layer of endocardial cells (Fig. 3A), and Elastin staining showed papillary fronds consisting of collagen and elastin fibers (Fig. 3B). The postoperative TTE and TEE showed no clear remnant tumor on the pulmonary valve. She was extubated after 5 h, recovered uneventfully, and was discharged on postoperative day 13. No recurrence on the pulmonary valve was observed during the follow-up one year after the operation.

## Discussion

Primary cardiac tumors are rare and account for 0.0017–0.33% of all cardiac tumors based on autopsy studies [3, 4]. Myxoma was thought to be the most common at 24–37%, followed by angiosarcoma (7.3–8.5%) and PFE (7.9–8.0%) [5]. Since 2003 when Gowda et al. reported the first case with PFE [6], studies have demonstrated that PFE was either originating from the aortic valve (44%), the mitral valve (35%), the tricuspid valve (15%), or the pulmonary valve (8%) [6, 7]. The etiology is unclear and requires further elucidation, but many of these cases are thought to be acquired [6]. Most patients with PFE are asymptomatic and were incidental finding during preoperative examinations [8]. Conversely, some cases with life-threatening cardiac and neurological symptoms such as cerebral infarction, transmit ischemic attack, myocardial infarction, and heart failure have also been reported in association with PFE because it typically originates in the left heart valves [9, 10]. In this case report, we highlight the importance of accurate diagnostic imaging of incidental PFE findings on the pulmonary valve so that they can be managed appropriately based on the recommendation from the available literature.

The development of high-resolution imaging, especially TEE, have facilitated rapid diagnosis of PFE in asymptomatic patients. TTE/TEE, CT, and MRI are thought to be effective diagnostic examinations of PFE, especially as the sensitivity and specificity of TTE are reported to be higher than other examinations with measures of 88.9% and 87.8%, respectively [11]. Indeed, contrast CT is inferior to TEE in terms of detecting a small mobile structure, and MRI is more useful than CT due to its advantages in soft tissue evaluation [12]. A few case series have prioritized curative surgical resection over pre-operative differential diagnosis of the tumor by various examinations because many symptomatic patients with PFE presented cardiac symptoms and required rapid surgical intervention to prevent further complications [13, 14]. In contrast, because our patient was an asymptomatic and non-urgent case, we were afforded time to diligently scrutinize the case by a relatively large number of examinations. In our patient, benign endocarditis papilloma was strongly suspected from the preoperative imaging evaluation by TEE and TEE and insight from previous studies; however, as endocarditis papilloma is rare in the pulmonary artery area and the tumor’s position along the pulmonary area was unclear, we also could not exclude the possibility of a malignant tumor attached to the wall of the pulmonary artery or determine its exact location even with various pre-operative examinations.

Currently, there is limited consensus with regards to the management of right-sided PPE. So far, asymptomatic patients with a non-mobile tumor have been managed conservatively, and then surgical resection was performed to prevent further complications when the patients presented any symptoms [6, 12]. Anticoagulant treatment was considered for high-risk patients [15]. In contrast, in patients with a pedunculated and highly-mobile tumor, tumor mobility was reported to be an independent predictor of cerebrovascular events and death [4, 8], suggesting that surgical resection is strongly recommended [14]. The surgical strategy for PFE resection generally requires valve repair or replacement in case of potential valve defect by massive tumorectomy, but most patients are managed by tumorectomy without valve repair or replacement. One report recommended surgical intervention in asymptomatic patients with incidental cardiac PFE [16]. Additionally, some literature reviews showed that successful complete resection of PFE induced significant long-term prognosis and lower stroke risk, while patients with suspected PFE without surgical intervention had high risk of cerebrovascular events and mortality [6, 7]. In our case, we performed planned tumorectomy and concomitant with AVR according to the above studies and intraoperative diagnosis. Nonetheless, it is also important to consider arguments against surgical intervention in these cases. In any case, thorough and proper examination may be necessary before surgical intervention to prevent fatal cardiac and cerebrovascular events and reduce mortality.

## Conclusion

This case provides valuable insight into a successful surgical treatment of pulmonary valve PFE without any complications, highlighting the importance and challenges of accurate diagnostic imaging.

## Data Availability

The datasets used are available from the corresponding author on reasonable request.
